# Constraint-Based Reconstruction and Analyses of Metabolic Models: Open-Source Python Tools and Applications to Cancer

**DOI:** 10.3389/fonc.2022.914594

**Published:** 2022-07-07

**Authors:** Rachel H. Ng, Jihoon W. Lee, Priyanka Baloni, Christian Diener, James R. Heath, Yapeng Su

**Affiliations:** ^1^ Institute for Systems Biology, Seattle, WA, United States; ^2^ Department of Bioengineering, University of Washington, Seattle, WA, United States; ^3^ Medical Scientist Training Program, University of Washington, Seattle, WA, United States; ^4^ Program in Immunology, Clinical Research Division, Fred Hutchinson Cancer Research Center, Seattle, WA, United States; ^5^ Herbold Computational Biology Program, Vaccine and Infectious Disease Division, Fred Hutchinson Cancer Research Center, Seattle, WA, United States

**Keywords:** cancer, metabolism, constraint-based modeling, genome-scale metabolic models, systems biology, omics, python, single-cell analysis

## Abstract

The influence of metabolism on signaling, epigenetic markers, and transcription is highly complex yet important for understanding cancer physiology. Despite the development of high-resolution multi-omics technologies, it is difficult to infer metabolic activity from these indirect measurements. Fortunately, genome-scale metabolic models and constraint-based modeling provide a systems biology framework to investigate the metabolic states and define the genotype-phenotype associations by integrations of multi-omics data. Constraint-Based Reconstruction and Analysis (COBRA) methods are used to build and simulate metabolic networks using mathematical representations of biochemical reactions, gene-protein reaction associations, and physiological and biochemical constraints. These methods have led to advancements in metabolic reconstruction, network analysis, perturbation studies as well as prediction of metabolic state. Most computational tools for performing these analyses are written for MATLAB, a proprietary software. In order to increase accessibility and handle more complex datasets and models, community efforts have started to develop similar open-source tools in Python. To date there is a comprehensive set of tools in Python to perform various flux analyses and visualizations; however, there are still missing algorithms in some key areas. This review summarizes the availability of Python software for several components of COBRA methods and their applications in cancer metabolism. These tools are evolving rapidly and should offer a readily accessible, versatile way to model the intricacies of cancer metabolism for identifying cancer-specific metabolic features that constitute potential drug targets.

## Introduction

Cancer involves a complex set of dysregulations in multiple biomolecular layers including metabolism. Metabolic changes in cancer result from and lead to profound changes in the behavior of cancer cells and their surrounding environment. Although extensively studied, these metabolic changes are difficult to accurately measure and model in an unbiased manner due to the need to consider a heterogeneous tumor environment encompassing different cell types, many difficult-to-measure metabolites, and lack of standardization of models (1). While recent years have yielded a wealth of methods to measure and analyze biological systems at multiple omics layers (genomic (2, 3), epigenomic (4), proteomic (5–8), and metabolomic (9–11), often extending to single-cell resolution (12), metabolic systems are difficult to systematically assess because gene expression or protein levels may not directly translate into metabolic activity (1).

**Figure 1:**
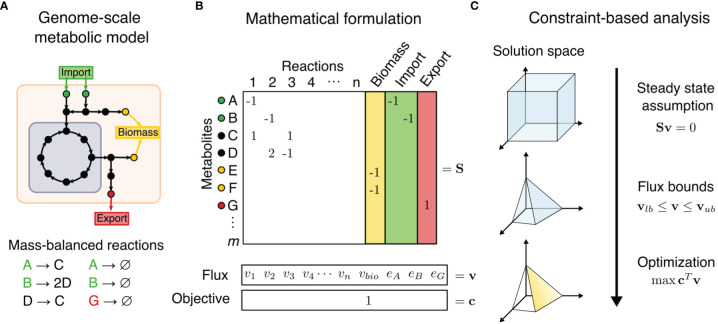
Constraint-based metabolic modeling. **(A)** A genome-scale metabolic model is a compartmentalized network of mass-balanced reactions that convert products to reactants, and boundary pseudo-reactions that import or export metabolites. Biological objectives, such as biomass production, require activity through a subset of internal reactions. **(B)** The metabolic model is converted into a stoichiometric matrix (S) of size *m* × *n*, with rows representing *m* metabolites and columns *n* reactions. Reaction flux through all internal reaction (*v_i_*) and exchange reactions (*e_i_*) is represented by vector v of length *n*. Objective function Z = c*^T^* v is formulated as a linear combination of desired fluxes, weighted by vector c. **(C)** At steady state, the rate of production and consumption of a metabolite must be zero, which is described by the system of equations Sv = 0. There are many solutions to this system of equations, but the solution space can be constrained by imposing flux bounds (v*_lb_* ≤ v ≤ v*_ub_*) and optimization such as maximization of objective function.

Genome-scale metabolic models (GEMs) can provide a compelling approach towards understanding cellular metabolism. GEMs are curated computational descriptions of entire cellular metabolic networks. Derived from genome annotations and experimental data, GEMs are composed of mass-balanced metabolic reactions and gene-protein associations that map the relationship of genes to proteins involved in each reaction (**Figure 1**). The accumulation of high-throughput data has contributed to the reconstruction of GEMs for hundreds of organisms, from microbes and model organisms to animals and humans (13). Whole-organism GEMs can further be reduced into context-specific and cell type-specific models for analyzing specific tissue phenotypic states performing different cellular functions. Metabolic flux analyses of GEMs have led to various model-guided applications, such as hypothesis generation, strain design, drug target discovery, multicellular interactions modeling, and disease etiology (14–16). With the rapidly increasing availability of high-resolution multi-omics datasets, there is an increasing need for tools to interpret data using a mathematical framework that also integrates existing vast and complex biological knowledge. In particular, dysregulated metabolic systems in cancer interact heavily with the surrounding environment, and metabolic flux analysis may prove especially beneficial to modeling these systems.

Compared to omics analysis, cancer metabolism may be more accurately modeled by combination of GEMs and a family of methods called Constraint-Based Reconstruction and Analysis (COBRA). COBRA methods perform systems-level analyses on metabolic networks to uncover how genetic and environmental factors affect phenotype on a biomolecular basis. COBRA framework utilizes a stoichiometric matrix that transcribes mass-balanced metabolic reactions of a cellular system, including the system’s uptake and secretion rates, into a matrix that represents the change in levels of reactants and products for each reaction (**Figure 1**). While there are many allowable states of reaction fluxes through a metabolic network, COBRA reduces this solution space of feasible flux distributions by adding constraints. Some basic constraints are mass conservation (stoichiometry of reaction and products in a reaction), steady-state assumption (input and output fluxes are balanced), and reaction flux bounds (inequalities of upper and lower bounds). Additional constraints can be determined by metabolite and enzyme levels, thermodynamics directionality, enzyme capacities, spatial compartmentalization, and genome regulatory mechanisms (15, 17). This induces a space of feasible fluxes which fulfill the used balance equations and constraints, often called the “flux cone”. Constraint-based analysis methods then aim to find biologically relevant flux distributions within the flux cone.

COBRA methods for metabolic network analysis are now incorporated into many software packages across several programming languages like MATLAB and Python (15). Of these, MATLAB packages such as COBRA Toolbox, Raven Toolbox, and CellNetAnalyzer have been the leading standard platforms that integrate with many existing COBRA methods (18–20). However, the reliance on MATLAB, a proprietary and closed-source software, reduces the accessibility of metabolic flux analysis, especially for teaching and reproducibility purposes. Recent open-source community efforts have promoted the development of a similar ecosystem of COBRA software in Python, starting with the development of COBRApy (21) under the openCOBRA Project (22) and PySCeS CBMPy (23). As an open-source language, Python opens COBRA methods to greater possibilities by enabling deployment on machines without a proprietary license, which is especially convenient for cloud computing. Due to Python being widely adopted for data science and computation, it provides state-of-the-art scientific tools for accessing databases, integrating various data modalities, and interfacing with computational tools like parallel computing, machine learning, visualizations, and web applications.

This review will summarize the set of packages currently available in Python for various COBRA methods. We identify the advantages and shortcomings of the Python ecosystem to guide users’ decisions on their choice of a software platform and inspire future research ideas. We focus on the application of COBRA methods to cancer metabolism. Finally, we will explore the future directions of COBRA methods development and their importance in cancer modeling.

## COBRA Methods in Python

**Figure 2:**
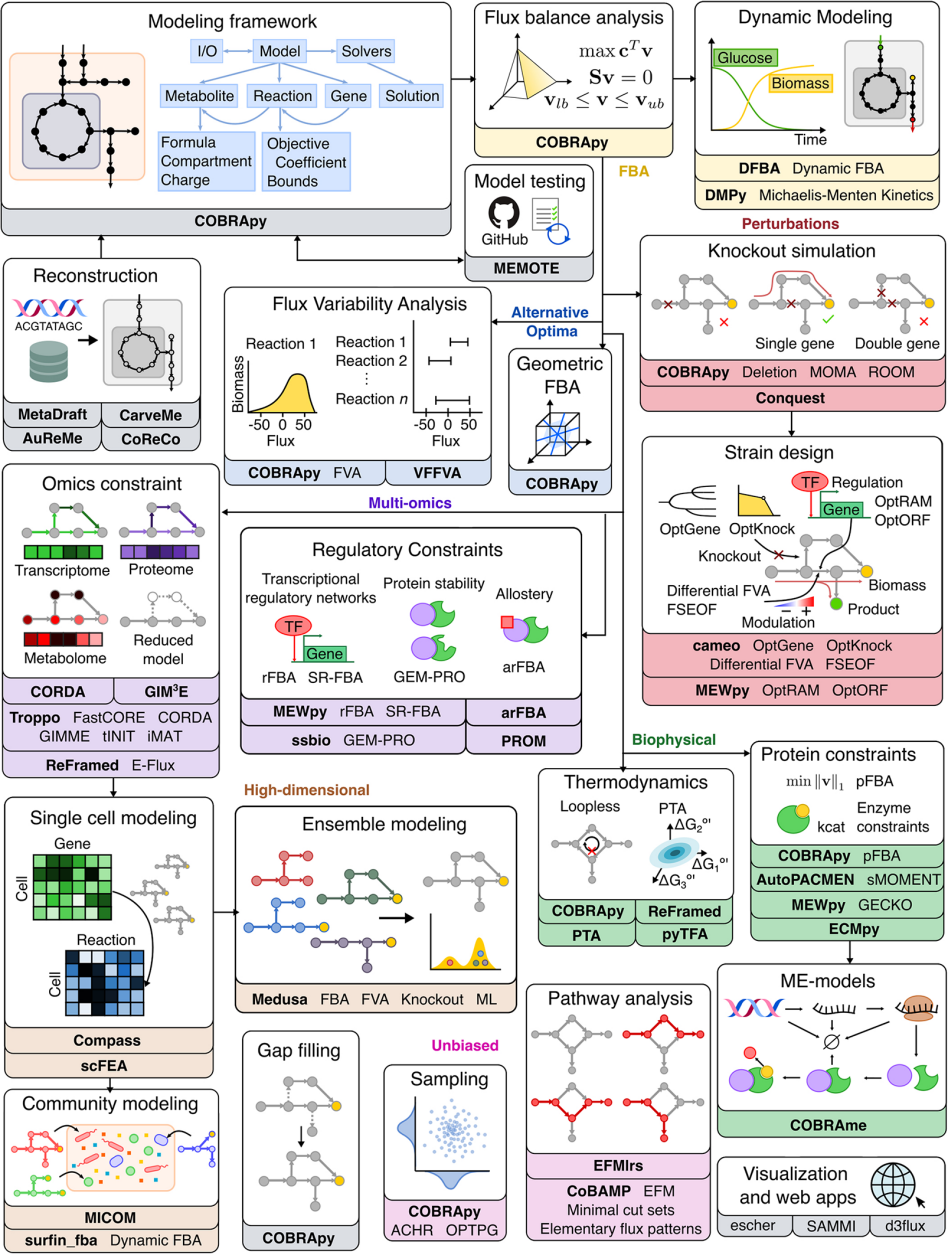
Overview of Python software for major components of COBRA methods. Constraint-based metabolic modeling first requires loading a metabolic model into software that handles the various parts of the modeling framework (grey), such as metabolites, reactions, genes, stoichiometric matrix, and flux solutions. New metabolic models can be reconstructed from genome sequences and database, quality-checked by model testing software, made consistent using gap-filling tools, and visualized using web-based packages. Using the metabolic model, FBA (yellow) finds an optimal flux distribution that follows stoichiometry under steady state and can further be extended to dynamic systems. Since there are alternative optima (blue) to FBA, FVA and geometric FBA can be used to characterize the solution space. We can perturb (red) the system to predict the effect of knockouts and use such predictions to design an optimal system (‘strain’). To improve FBA predictions, we can add biophysical (green) constraints based on thermodynamics, proteins, and macromolecular expression. Metabolic modeling can be further enhanced by integration of multi-omics (purple) data, such as extracting reduced models based on omics data and adding regulatory constraints. Using omics data, metabolic modeling can become high-dimensional (brown), through single cell modeling and community modeling. Multiple metabolic models can be reduced into ensemble objects. In contrast to FBA, unbiased (pink) approaches do not require an objective function. These include methods for sampling flux distributions and pathway analyses. Names of software packages are in bold.

To make COBRA open-source and accessible, multiple Python packages have been developed by the scientific community to perform the different analyses within COBRA. Here we describe the major components of COBRA and list their associated packages (**Figure 2**; **Table 1**), and assess their strengths and weaknesses (**Table 2**). First, we start with the core package COBRApy, which handles the details of metabolic models and basic simulations. We then describe methods for determining metabolic flux, such as flux balance analysis, flux variability analysis, and *in silico* perturbation. Next, we summarize various methods for adding biological constraints like multi-omics and biophysics. In addition, we review methods for unbiased pathway analysis and sampling methods. We also summarize the development of COBRA methods for models at the single-cell and population level. Finally, we touch upon packages for visualization and interactive web applications.

**Table 1:** Python tools for constraint-based modeling.

Category	Method	Software	URL	Doc.
Modeling framework	Object-oriented programming	COBRApy (21)	https://cobrapy.readthedocs.io https://github.com/opencobra/cobrapy	✔
	Testing	MEMOTE (24)	https://memote.readthedocs.io https://github.com/opencobra/memote	✔
Reconstruction	Template-based	AuReMe (25)	https://aureme.readthedocs.io http://aureme.genouest.org	✔
	Template-based, gap-filling	CarveMe (26)	https://carveme.readthedocs.io https://github.com/cdanielmachado/carveme	✔
	Template-based	MetaDraft (27)	https://systemsbioinformatics.github.io/cbmpy-metadraft/	✔
	Homology-based, multi-species, gap-filling	CoReCo (28)	https://github.com/esaskar/CoReCo	✔
FBA	FBA (29)	COBRApy	See above	✔
Dynamic metabolic modeling	Dynamic FBA (30)	dfba (31)	https://dynamic-fba.readthedocs.io https://gitlab.com/davidtourigny/dynamic-fba	✔
	Michaelis-Menten kinetics	DMPy (32)	https://gitlab.com/wurssb/DMPy	✔
Alternative optima	Geometric FBA (33)	COBRApy	See above	✔
	FVA (34)			
	VFFVA	VFFVA (35)	https://vffva.readthedocs.io https://github.com/marouenbg/VFFVA	✔
Knockout Simulation	Single/Double deletions (36)	COBRApy	See above	✔
	MOMA (37)			
	ROOM (38)			
	Flux- and graph-based	Conquest (39)	https://github.com/laniauj/conquests	✔
Strain Design	OptGene (40)	Cameo (41)	https://cameo.bio/ https://github.com/biosustain/cameo	✔
	OptKnock (42)			
	Differential FVA			
	FSEOF (43)			
	OptRAM (44)	MEWpy (45)	https://mewpy.readthedocs.io https://github.com/BioSystemsUM/mewpy	✔
	OptORF (46)			
Omics constraints	E-flux (47)	ReFramed (48)	https://reframed.readthedocs.io https://github.com/cdanielmachado/reframed	✔
	CORDA	CORDA (49)	https://github.com/resendislab/corda	✔
	GIM^3^E	GIM^3^E (50)	https://github.com/brianjamesschmidt/gim3e	✔
	FASTCORE (51)	Troppo (52)	https://github.com/BioSystemsUM/troppo	✘
	CORDA (49)			
	GIMME (53)			
	tINIT (54)			
	iMAT (55)			
Regulatory constraints	rFBA (56)	MEWpy	See above	✔
	SR-FBA (57)			
	PROM	PROM (58)	https://github.com/jseidel5/Python-Probabilistic-Regulation-of-Metabolism	✔
	GEM-PRO (59)	ssbio (60)	https://ssbio.readthedocs.io	✔
	arFBA	arFBA (61)	https://github.com/cdanielmachado/arfba	✘
Thermodynamics	ll-FBA (62)	COBRApy	See above	✔
	CycleFreeFlux (63)			
	PTA	PTA (64)	https://probabilistic-thermodynamic-analysis.readthedocs.io https://gitlab.com/csb.ethz/pta	✔
	TFA, TVA (65)	ReFramed	See above	✔
	TFA, TVA (65)	pyTFA (66)	https://pytfa.readthedocs.io https://github.com/EPFL-LCSB/pytfa	✔
Protein constraints	pFBA (67)	COBRApy	See above	✔
	GECKO (68)	MEWpy	See above	✔
	sMOMENT	AutoPACMEN (69)	https://github.com/klamt-lab/autopacmen	✔
	ECMpy	ECMpy (70)	https://github.com/tibbdc/ECMpy	✔
ME-modeling	COBRAme	COBRAme (71)	https://cobrame.readthedocs.io	✔
Gap filling	MILP	COBRApy	See above	✔
Ensemble modeling	FBA	Medusa (72)	https://medusa.readthedocs.io/ https://github.com/opencobra/Medusa	✔
	FVA			
	Deletion			
	ML			
Single cell modeling	Compass	Compass (73)	https://yoseflab.github.io/Compass/ https://github.com/YosefLab/Compass	✔
	scFEA	scFEA (74)	https://github.com/changwn/scFEA	✔
Community modeling	MICOM	MICOM (75)	https://micom-dev.github.io/micom/ https://github.com/micom-dev/micom	✔
	Dynamic FBA	surfin_fba (76)	https://github.com/jdbrunner/surfin_fba	✔
Sampling	ACHR (77)	COBRApy	See above	✔
	OPTPG (78)			
Pathway analysis	EFM	EFMlrs (79)	https://github.com/BeeAnka/EFMlrs	✔
	EFM (80)	CoBAMP (81)	https://cobamp.readthedocs.io https://github.com/BioSystemsUM/cobamp	✔
	Minimal cut sets (82)			
	Elementary flux patterns (83)			
Visualization and web apps	Plug-in, website	Escher (84)	https://escher.readthedocs.io https://escher.github.io	✔
	Plug-in, website	SAMMIpy (85)	https://sammipy.readthedocs.io www.SammiTool.com	✔
	Plug-in	d3flux (86)	https://pstjohn.github.io/d3flux/ https://github.com/pstjohn/d3flux	✔

Methods and their associated software packages as illustrated in **Figure 2**, organized by their general function. Weblinks to each software’s official website and documentation are provided if available. Each software is assessed for availability of documentation (Doc.) or any form of demonstrative examples.

**Table 2:** Pros and cons of COBRA methods.

Category	Method/Tool	Pros	Cons
**Reconstruction**	AuReMe	- Support for eukaryotes model - Good traceability - Automatic integration of experimental data	- Some manual refinement assistance - Not FBA-ready
	CarveMe	- GEMs ready for FBA - Fast - Customizable for large number of genomes	- No manual refinement assistance - Some support for eukaryotes model
	MetaDraft	-Support for eukaryotes model - Fast	- No manual refinement assistance - Not FBA-ready
	CoReCo	- Support for eukaryotes model - GEMs nearly ready for FBA - Simultaneous reconstruction for multiple species (parallelizable)	- Requires KEGG license - No manual refinement assistance
**FBA**	FBA	- Does not require kinetic parameters	- Requires objective function - Requires reaction bounds (especially exchange flux)
**Dynamic modeling**	Dynamic FBA (SOA and DAE)	- Couples pseudo-steady states to dynamical systems - Does not require kinetic parameters	- SOA requires small steps and thus more computation
	DMPy	- Infers missing kinetic parameters using thermodynamics constraints	- Requires >80% of kinetic parameters for accuracy
**Alternative optima**	Geometric FBA	- Gives single representative solution – Reproducible typical solution (avoids randomly picking one solution from flux cone)	- Weak correlation with protein levels (without omics constraint)
	FVA/VFFVA	- Determines min and max flux for a reaction would achieve optimal objective state - (VFFVA) Increased speed and reduced memory usage	- Varies one reaction at a time
	Sampling	- Estimates probability distribution of feasible fluxes - Can be unbiased (not using an objective function)	- Computationally intensive
**Omics constraints**	E-flux	- Constraints reaction bounds only - No discretization of data	- May over-constrain model based on noisy data - Poor growth rate prediction
	GIMME	- LP problem (fast) - Ensures operability of required metabolic function - Predicts growth rate, uptake/secretion rates, essential genes, and oncogenes	- Discretizes data - Models have high fractions of blocked reactions, moderate resolution power, poor robustness to missing data/noise
	GIM^3^E	- Ensures operability of required metabolic function - Integrates metabolomics data	- Discretizes data - MILP problem (slow)
	(t)INIT	- Ensures operability of required metabolic functions - (INIT) predicts oncogenes and tumor suppressor genes, consistent model, good resolution power, robust to noise/missing data	- MILP problem (slow) - (INIT) Poor predictions of growth rate, uptake/secretion rates, and essential genes
	iMAT	- No objective required - Consistent model, good resolution power, robust to noise/missing data - Predicts oncogenes	- Discretizes data - MILP problem (slow) - Weak predictions of growth rate, uptake/secretion rates, and essential genes
	FASTCORE	- LP problem (fast) - Obtains minimal consistent model - Predicts oncogenes and loss of function mutations - Moderately consistent model, good resolution power, robust to noise	- Requires specification of core reactions - Poor predictions of growth rate, uptake/secretion rates, and essential genes
	CORDA	- LP problem (fast) - Non-parsimonious pruning - Predicts oncogenes and loss of function mutations	- Requires specification of core reactions - Weak predictions of growth rate and essential genes - Poor predictions of uptake/secretion rates
**Regulatory constraints**	rFBA	- Predicts flux over time intervals - Models transcriptional regulation	- Uses boolean TRN - Stepwise calculation of metabolic and regulatory states - Chooses only one solution per time interval
	SR-FBA	- Combined calculation using metabolic and regulatory constraints - Models transcriptional regulation	- Uses boolean TRN - Calculates flux for one time step (steady-state) - Does not account for metabolic transitions and feedback loops
	PROM	- Uses continuous TRN - Models transcriptional regulation	- Requires TF-target gene relationships
	GEM-PRO	- Models protein instability	- Requires protein structures
	arFBA	- Models allosteric regulation	- Requires regulation matrix defining effector-reaction relationship - Small-scale applications
**Thermodynamics**	ll-FBA	- Does not require metabolite concentrations or free energies	- MILP problem (slow)
	CycleFreeFlux	- Post-process using LP problem (fast) - Can be applied to any flux distribution including sampled solutions - Does not require metabolite concentrations or free energies	- Biased towards solutions with small total flux and those with same direction as their overlapping internal cycles
	TFA, TVA	- Explicitly models thermodynamics	- Requires metabolite concentrations and free energies - Over-approximates uncertainty
	PTA	- Explicitly models thermodynamics for optimization and sampling - Models uncertainty of free energies and metabolite concentrations	- Requires metabolite concentrations and free energies - Computationally intensive
**Protein constraints**	pFBA	- Predicts growth rate, uptake/secretion rates, and essential genes	- Assumes that flux distribution with smallest magnitude minimizes protein costs
	Enzymatic constraints (GECKO, sMOMENT, ECMpy)	- Model proteome limitation at enzyme resolution - (sMOMENT) Automates enzyme database query - (ECMpy) Automates enzyme parameters calibration - (ECMpy) Does not increase model size	- Requires experimentally measured enzyme turnover numbers - (GECKO) Increases model size - (sMOMENT) Moderately increases model size - (ECMpy) Manually obtains protein subunit composition data
**ME-modeling**	COBRAme	- Modeling proteome composition improves predictive accuracy - Framework for building ME-models for new organisms	- Large model size and complexity - No standardized SBML format for ME-models - Only applied to bacteria so far
**Ensemble modeling**	Medusa	- Compresses multiple models into compact ensemble objects - Reduces memory usage of storing ensembles - Interfaces with machine learning	- No standardized SBML format for ensemble objects
**Single cell modeling**	Compass	- Genome-scale modeling - Maximizes agreement with gene expression - Handles sparsity by sharing information across neighbors - Uses multiple objective functions	- Map gene expression to reaction expression using boolean relationships (GPR)
	scFEA	- Minimizes flux imbalance of all cells to simulate exchange of metabolites - Less stringent flux balance and steady-state assumption - Uses neural net to model nonlinear relationship between gene expression and reaction rates	- Not easily scalable due to large memory usage - Applied to small-scale models
**Community modeling**	MICOM	- Models exchanges and interactions between communities and environment - Automates building community models from a model database - Predicts replication rates in human gut microbiome	- Assumes trade-offs between individual and community growth rate (gut microbiome specific) - Metabolic models may not be accurate (labratory vs. gut conditions, species differences)
	Dynamic FBA (surfin_fba)	- Reduces optimizations problems (and parameter space) required for dynamic FBA for communities	- Non-biological approach to choosing between non-unique optima
**Pathway Analysis**	EFM	- Unbiased characterization of models (no objective function required) - (EFMlrs) Pre- and post-process models for EFM calculations	- (EFMlrs) EFM calculation performed by other tools not included in program - EFM calculations are memory intensive and not scalable

Some method comparisons extracted from literature for reconstruction (87, 88), dynamic modeling (89), omics constraints (90, 91), and regulatory constraints (92). Growth rate, uptake/secretion rates, and cancer essential gene prediction performances from Jamialahmadi et al. are based on human metabolic models and available only for GIMME, INIT, iMAT, FASTCORE, CORDA, and pFBA (91).

### Modeling Framework

COBRA for Python (COBRApy) uses an object-oriented programming approach to represent models, metabolites, reactions, and genes as class objects with accessible attributes. Using this design, COBRApy recapitulates functions for standard metabolic flux analyses of its MATLAB counterpart while being extendible and accessible. First, it has the capabilities to read and write models in various formats such as MAT-file (for storing MATLAB variables), JSON, YAML, and Systems Biology Markup Language (SBML) (93), the current community-accepted standard for computational systems biology. SBML incorporates the Flux Balance Constraints (FBC) version 2 package (94), which supports constraint-based modeling by encoding objective functions, flux bounds, model components, and gene-protein associations, whose usage will be discussed below. COBRApy can also load SBML models from web databases such as BiGG and BioModels (95, 96). The quality of such metabolic models can be assessed using a Python test suite called MEMOTE that integrates version control of models *via* GitHub and checks for correct annotation, model components, and stoichiometry (24). To use these models for various optimization problems, COBRApy interfaces with either commercial or open-source solvers that implement linear programming algorithms. We will detail additional built-in or integrated functionalities for various COBRA methods (**Figure 2**).

### Flux Balance Analysis

The most common COBRA method is flux balance analysis (FBA), which assumes the system is at steady state, follows mass-balance described in the stoichiometric matrix, and restricts reaction fluxes by bounds. Furthermore, FBA searches for sets of steady-state reaction fluxes that maximize or minimize an objective function representing a biological function, such as using biomass production objective to model cellular growth (29). The objective function is an artificial reaction formulated by linear combinations of reactions that would contribute to the desired biological function. For example, the biomass production can be represented by the consumption of biomass precursors in different proportions. Components of the biomass production may include amino acids, lipids, nucleotides, carbohydrates, cofactors, and other molecules based stoichiometrically on the macromolecular composition of a cell measured as weight fractions under specific experimental conditions, typically during exponential growth. Although the biomass equation is the de facto choice for the objective function and macromolecular compositions are more similar across related species, certain components such as fatty acids are sensitive to environmental and genetic conditions (97). Therefore, caution is required when choosing an appropriate objective function that reflects the system’s experimental condition. Sensitivity analysis of FBA could be performed using different objectives (or ensemble of objectives) accounting for the natural variation in biomass equation across different conditions (97). Assessment of bias introduced by the objective function would require experimental validation of growth dynamics or knockout simulations discussed below. Under the above mathematical constraints, FBA is an optimization problem involving a system of equations that can be solved by linear programming, as initially proposed in 1984 (98). Functions for FBA and customization of objective functions are included in COBRApy. With these basic constraints, FBA is the foundation from which many forms of COBRA methods evolved.

### Dynamic Metabolic Modeling

Although FBA assumes that a system is unchanging at steady state, these pseudo-steady states can be coupled to a dynamical system with changing environmental variables using dynamic FBA (DFBA) (99). There are several approaches to DFBA: 1) dynamical optimization approach (DOA) that uses ordinary differential equations (ODEs) to describe an optimization problem of entire time profiles of metabolites, 2) statistic optimization approach (SOA) that divides the time period into time intervals to perform instantaneous optimization (LP) per time interval with flux rate-of-change constraints, 3) direct approach (DA) that resolves the LP of the right-hand side of ODEs, and 4) reformulation of the ODEs as differential-algebraic equation (DAE) system (30, 89, 99). The fourth approach via DAE is implemented in Python package dfba (31), while the second approach *via* SOA can be implemented using COBRApy and SciPy. Alternatively, a very different approach to dynamical metabolic modeling was proposed by DMPy, which translates a GEM into a dynamic reaction equation model using Michaelis-Menten approximations and infers missing kinetic constants using Bayesian parameter estimation with thermodynamics constraints (32). However, this method requires extensive measurements of reaction rates to accurately parameterize a large-scale model. All these constraint-based methods for dynamical metabolic modeling enable the utilization of high-throughput and longitudinal data to interrogate changes in metabolism.

### Alternative Optimal Solutions

Flux distributions, even under an optimal objective, are usually not unique as many alternative fluxes can yield a maximum biomass production. The most representative solution can be found using geometric FBA in COBRApy, which looks for a unique flux distribution that is central to the entire solution space (33). To better characterize all alternative optima that satisfy the constraints of FBA, flux variability analysis (FVA) finds the range of alternative fluxes for a reaction that maintains optimization of the objective function within a margin of error (34). The search for alternate optimal solutions is time-intensive, but COBRApy has addressed this problem in FVA by implementing parallel computing. For example, Very Fast Flux Variability Analysis (VFFVA) is available in Python and its implementation of FVA is much faster and more memory-efficient than its analog in MATLAB, fastFVA (35).

### System Perturbations, *In Silico* Knockout, and Strain Design

Quantitative flux predictions are useful to experimentalists because of their potential to explain or even predict the effect of environmental and genetic changes. For investigating the relationship between the external environment and the modeled system, COBRApy provides tools for specifying the growth medium and exchange rates of a model. Instead of extracellular conditions, intracellular changes such as genetic mutations and gene modulation can be interrogated as well. To identify essential genes and reactions for biological functions, FBA is performed with gene knockout simulations to assess the effects of the knockouts on objective functions (36). Similar to COBRA Toolbox, COBRApy includes functions for knocking out single or double genes and reactions by restricting the flux through associated reactions. Another algorithm for assessing the effect of a perturbation is minimization of metabolic adjustment (MOMA), which determines the post-perturbation flux vector that is closest to a reference flux vector (e.g., FBA solution before change) (37). Currently, COBRApy implementation of MOMA is the only one that does not require a commercial quadratic programming solver but instead uses OSQP, which is an open-source solver (100). Another method, called Regulatory-on-off minimization (ROOM), finds the new flux distribution with minimal reaction changes compared to a reference state (38). Available in COBRApy, these methods characterize the effects of gene deletion relative to a wild-type reference. Adding to flux-based determination of essentiality, a new metabolite essentiality analysis combining graph-based and flux-based analysis was proposed by Conquests (Crossroad in metabOlic Networks from Stoichiometric and Topologic Studies) (39).

The iterative testing of gene or reaction deletions was initially developed for *in silico* strain design, which determines optimal genetic changes that would maximize production of desired metabolites. Straight maximization of only the desired reaction is problematic, since it ignores the drainage of cellular resources needed for cellular growth. Therefore, strain design methods couple product yields with cellular objectives to optimize for fast-growing cells that have high productivity. Such metabolic engineering tools are available in a COBRApy-derived package called cameo (41). It provides efficient, parallelized implementations of standard *in silico* strain design methods for predicting gene knockout strategies (OptGene [evolutionary algorithm] (40), OptKnock [linear programming] (42) and for predicting gene expression modulation targets (Differential FVA, Flux Scanning based on Enforced Objective Flux [FSEOF] (43). Instead of modulating genes, there are algorithms that optimize at the regulatory level by changing transcription factors, such as OptRAM (44) and OptORF (46) in MEWpy (Metabolic Engineering Workbench in python) (45). These simulation tools for strain design and *in silico* knockouts/perturbations can be easily adapted to study metabolism in the context of physiology and disease, especially cancer. For example, we will later discuss studies that use *in silico* knockout to screen for cancer drug targets. Other studies integrated genetic variants by simulating knock out of enzymes with loss of function mutations (101–103).

### Integrating Multi-Omics Data With GEMs

Integration of omics data into metabolic models is now critical to standard analysis of GEMs to improve flux predictions and interpret multi-omics data. Prior to applying constraints, gene-level data must first be processed to reflect reaction-level data. This involves calculating a reaction expression matrix that evaluates gene-protein-associations (GPR, nested logic rules representing gene essentiality and redundancy). For example, we take the minimum expression of required subunits, but take the sum of isozyme expression. This calculation can be performed in Python packages like CORDA (Cost Optimization Reaction Dependency Assessment) (49) and MEWpy (45). Marín de Mas et al. further improved GPR evaluation in their Python implementation of stoichiometric GPR (S-GPR) that considers the stoichiometry of protein subunits (104).

The resulting reaction expression levels are used subsequently to extract a context-specific metabolic model of active reactions from the whole-organism GEM to reflect a phenotypic state specific to cell type and condition, such as disease state or nutrient level. The simplest transcriptome constraints can be applied by setting associated expression levels as the reaction upper bound, as demonstrated in E-flux and other studies (47, 105, 106). Instead of constraining all genes, PRIME is method that adjusts reaction upper bounds of phenotype-associated genes that are correlated with phenotypic data such as growth rate (90). Additional methods for extraction of context-specific models from transcriptome, metabolome, and proteome have been reviewed previously and can be summarized into three main families of approaches (107): 1) GIMME-like (GIMME (53), GIM^3^E (50), tINIT (54)), which aims to maximize the correspondence of flux phenotype to data while maintaining required metabolic functions; 2) iMAT-like (iMAT (55), INIT (108), Lee-12 (109), which only maximizes similarity of flux phenotype to data; and 3) MBA-like (MBA (110), mCADRE (111), FASTCORE (51), FASTCORMICS (112), CORDA (49), which removes non-core reactions while ensuring consistency of the model. Currently, integration of these methods with COBRApy is still in development within the DRIVEN project (113). Fortunately, some of these reconstruction methods have been reimplemented in other Python packages (**Table 1**). For example, ReFramed implemented E-flux (48), CORDA and GIM^3^E have standalone Python packages, and Troppo implemented FASTCORE, CORDA, GIMME, tINIT, and iMAT (52). Nonetheless, the Python ecosystem has shortcomings in reconstruction methods, such as the unavailability of some methods (INIT, MBA, mCADRE, FASTCORMICS, and PRIME), and the lack of documentation and usage examples for the Troppo package.

Reconstruction methods could result in incomplete and infeasible networks, partly due to errors in experimental data and curated knowledge, and partly due to parsimonious approaches when pruning reactions. To make reconstructed models feasible, one can use the gap-filling functionality in COBRApy to infer missing pathways using mixed-integer linear program (MILP). However, due to stochasticity and existence of alternative optima, GEM reconstruction and gap-filling of the same network can give rise to multiple GEMs that could yield different flux predictions. To account for the uncertainty in network structure, ensemble modeling compresses such a set of alternative models into an ensemble object to reduce redundancy while capturing variation. Ensemble modeling can be performed through Medusa, a Python package for generating ensembles, performing ensemble simulations, and coupling ensembles with machine learning (ML) (72).

Despite reconstruction of context-specific GEMs, GEMs are still flawed in flux prediction due to their inability to account for cellular mechanisms that regulate metabolic activity. A recent review has outlined the major methods for integrating regulatory mechanisms into metabolic models as the following: transcriptional regulatory networks (TRNs), post-translational modifications, epigenetics, protein–protein interactions and protein stability, allostery, and signaling networks (92). Several methods using TRNs have been translated from MATLAB to Python (**Table 1**), including boolean TRN methods like regulatory FBA (rFBA) (56) and steady-state regulatory FBA (SR-FBA) (57) available *via* MEWpy, and a continuous TRN method called probabilistic regulation of metabolism (PROM) (58, 114). Other regulatory mechanisms are also available: 1) GEM-PRO (59) integrates protein structure information, and 2) arFBA (61) integrates allosteric interactions respectively. However, methods for integrating post-translational modifications, epigenetics, and signaling networks are not yet available in Python. Future development is needed to account for the complex cellular regulatory activity.

Extraction of context-specific GEMs requires a reference GEM that is often manually curated. To automate the laborious process of GEM reconstruction, several tools were developed to reconstruct microbial GEMs from genome sequences (87). Several examples of Python-based software are AuReMe (25), CarveMe (26), MetaDraft (27), and CoReCo (28). Among these, Mendoza et al. (87) reviewed the first three and found them all to generate GEMs that have high reaction sets similarity to manually curated models, but only CarveMe generates GEMs ready-to-use for FBA (**Table 2**). A more recent tool called gapseq (88) was shown to outperform CarveMe, but it is written in shell-script and R.

### Biophysical Constraints

To ensure that reaction directionalities in computational results agree with biological findings, COBRA methods include addition of thermodynamic constraints *via* removal of thermodynamically infeasible pathways or calculations of Gibbs free energy. The vastness of solution space can also be attributed to thermodynamically infeasible loops where metabolites are cycled infinitely. COBRApy includes two implementations for removing such loops: one method ll-FBA (add_loopless) utilizes mixed-integer linear programming (62), and another faster method CycleFreeFlux (loopless_solution) uses postprocessing of solutions (63). Additionally, there are other Python packages that interface with COBRApy to implement thermodynamics analysis. For example, probabilistic thermodynamics analysis (PTA) models use joint probability distributions of free energies and concentrations for stream optimization and sampling flux analysis (64). Earlier methods such as thermodynamic flux analysis (TFA) and thermodynamic variability analysis (TVA) (65) were implemented in ReFramed (48). Another Python package for thermodynamic-based flux analysis (pyTFA) couples thermodynamics feasibility into FBA calculations (66). Thermodynamics constraints ensure physiological flux predictions and help to reduce the solution space.

Another theme of biophysical constraints involves modeling the proteome limitation of a cell due to molecular crowding in a cell. A simple method within this theme is parsimonious FBA (pFBA), which assumes that minimizing overall total flux approximately finds efficient pathways that minimizes the total enzyme mass (67). Available in COBRApy, pFBA first determines the maximum value of the objective function, then adds it as a model constraint and solves for the flux distribution with the smallest magnitude, minimizing protein costs (67). However, this assumption may not always hold for all conditions and complex cellular networks. Another way to limit proteins is to add constraints based on enzyme parameters such as turnover number (k_cat_) and molecular weight. These protein allocation constraints are applied by Python package MEWpy using a method called GECKO (Genome-scale model enhancement with Enzymatic Constraints accounting for Kinetic and Omics data), which adds many pseudo-metabolites and pseudo-reactions to represent enzymes (68). Another package for protein allocation constraints is AutoPACMEN (Automatic integration of Protein Allocation Constraints in MEtabolic Networks) (69). AutoPACMEN can automate database query and creation of models using sMOMENT (short metabolic modeling with enzyme kinetics), which introduces only one pseudo-reaction and pseudo-metabolite. Further improving upon these methods, ECMpy adds enzyme constraints without increasing model size (70). Studies have shown that adding protein constraints improves the accuracy of flux predictions by explaining suboptimal overflow metabolism and metabolic switches (69, 70). Instead of high-level protein constraints, the machinery cost of protein expression can be explicitly modeled using genome-scale models of metabolism and macromolecular expression (ME-models). ME-models extend GEMs by computing optimal composition of macromolecules like proteins, nucleotides, and cofactors, to model the entire process from transcription and translation, to complex formation and metabolic reaction. Software for building and simulating ME-models is currently only available in Python *via* COBRAme (71) and was extended to dynamic systems *via* dynamicME (115). All packages for protein constraints mentioned above are compatible with COBRApy.

### Unbiased Characterization of Solution Space

There are unbiased methods for analyzing distribution of steady-state flux through a metabolic model. One set of unbiased methods performs network-based pathway analysis without knowledge of traditional pathway annotations: elementary flux mode (EFM) analysis finds the minimum reaction sets (i.e., pathways) that can maintain steady state. Different variations of EFM have been implemented in Python. For example, EFMlrs is a Python package that performs EFM enumeration *via* lexicographic reverse search, an implementation that significantly improves performance and memory usage (79). In addition, CoBAMP is another package that has implemented EFM (80), minimal cut sets (82), and elementary flux patterns (81, 116). Extreme pathway (ExPa) analysis is another method for identifying reaction sets but it is not currently available in Python (83).

Another set of unbiased methods is Markov chain Monte Carlo (MCMC) sampling methods, which can characterize the solution space by estimating the probability distribution of feasible fluxes. This could be performed with or without constraining by an objective function. Currently, COBRApy integrated MCMC methods such as artificial centering hit-and-run (ACHR) (77) and optimized general parallel (OPTPG) (78) samplers, but not coordinate hit-and-run with round (CHRR) (117) that was found to be the best performing (118).

### Single-Cell Metabolic Modeling

Our ability to interrogate the heterogeneity of cell populations has grown rapidly due to advances in single-cell technologies that can measure the transcriptome, proteome, epigenome, and even metabolome at the single-cell level (2–7, 11, 12, 119–126). While single cell multi-omics data can be analyzed by pathway enrichment, clustering, and correlation methods (16, 122, 123), recent studies have developed algorithms in Python to calculate metabolic flux from single-cell transcriptome (119, 127). Zhang et al. demonstrated the usage of CORDA for the reconstruction of cell type-specific metabolic models from murine single-cell transcriptome and their subsequent FBA simulations of NAD^+^ biosynthesis using COBRApy (128). Instead of optimizing for a specified objective function, Compass is an FBA-based method that scores the ability of cell transcriptome to maintain high flux through each reaction (73). Rather than using linear programming to solve for flux distribution, scFEA first reconstructs a metabolic model into a directed factor graph, then trains a deep neural network to learn metabolic flux distributions by minimizing flux imbalance across all cells and maximizing correspondence with gene expression (74). Due to drop-outs in single-cell RNA-seq, these algorithms took different approaches to handle the sparsity of expression data: 1) Zhang et al. calculated mean expression profiles per tissue and cell ontology class, 2) Compass allows information sharing between cells that are similar in transcriptional space, and 3) scFEA trains the model on all cells and removes metabolic modules only if they are entirely composed of significantly unexpressed genes. These methods allow metabolic flux interpretation of single-cell transcriptome at the single-cell resolution; however, not all flux estimation methods account for the interaction of cells *via* uptake and secretion of metabolites into the environment.

### Multicellular Metabolic Modeling

To account for metabolic interactions, multicellular modeling was devised to model interplay between multiple metabolic networks coming from different species or tissues, with applications from microbiology to human physiology (129). Community modeling of the human gut microbiome reveals community-level function and cross-feeding interactions, as demonstrated by Python package MICOM (75). Community models are further extended using dynamic FBA of microbial communities, which can be efficiently calculated using Python package called surfin_fba that reduces the number of optimization timesteps when modeling communities (76). Early attempts to model human cell populations were explored using MATLAB, beginning with popFBA that simulated clones of cancer cells with identical stoichiometry and capacity constraints while allowing extracellular fluxes (130). PopFBA searched for combinations of individual metabolic flux distributions that would maximize a population object, e.g., total biomass, to explore metabolic heterogeneity and cooperation between single cells. However, this method gives many possible solutions and ignores the differences in metabolic requirements, functions, and proliferation rates of heterogeneous populations. To address both issues, single-cell FBA (scFBA) in MATLAB optimizes individual objective functions within a multi-scale model constrained by single-cell transcriptome and bulk extracellular fluxes to reduce the solution space (131). Overall, the added complexity of multicellular modeling can improve our interpretation of omics data and provide insights into cell-cell interactions important to many biological systems.

### Visualization and Web Application

While algorithm development for COBRA is important, the utility of COBRA methods also depends on the usability and dissemination of scientific results. Python libraries have enabled the development of more interactive, user-friendly applications for analysis and visualization of metabolic networks. For example, Escher is a web application for visualizing metabolic models and also a Python package with interactive widgets for Jupyter Notebooks that can visualize COBRApy models (84). Escher has been integrated into other Python COBRA packages such as cameo to visualize flux analysis results. Additional interactive visualization packages include SAMMI for semi-automated visualization and d3flux for d3.js based plots (85, 86). Due to open-source nature of Python packages, future COBRA web applications can be deployed for public use without licensing limitations.

## Genome-Scale Modeling of Cancer Metabolism With COBRA Tools

Cancer cells undergo metabolic reprogramming to promote proliferation and invasion, and in turn alter the nutrient-levels and cell types within the tumor microenvironment (TME). We here summarize these metabolic changes and provide the rationale for using COBRA methods to analyze cancer metabolism and TME. Indeed, COBRA methods have been utilized for various applications in cancer research in the past decades. We describe how the analyses begin with building cancer-specific metabolic models, from which one can infer metabolic dysregulation through pathway and network analyses. Next, we showed how these models were used for quantitative prediction of cancer metabolic activity and drug targets. Finally, we highlight the frontiers of modeling the TME using multicellular or single-cell COBRA methods.

### Metabolism of Cancer and the Tumor Microenvironment

**Figure 3:**
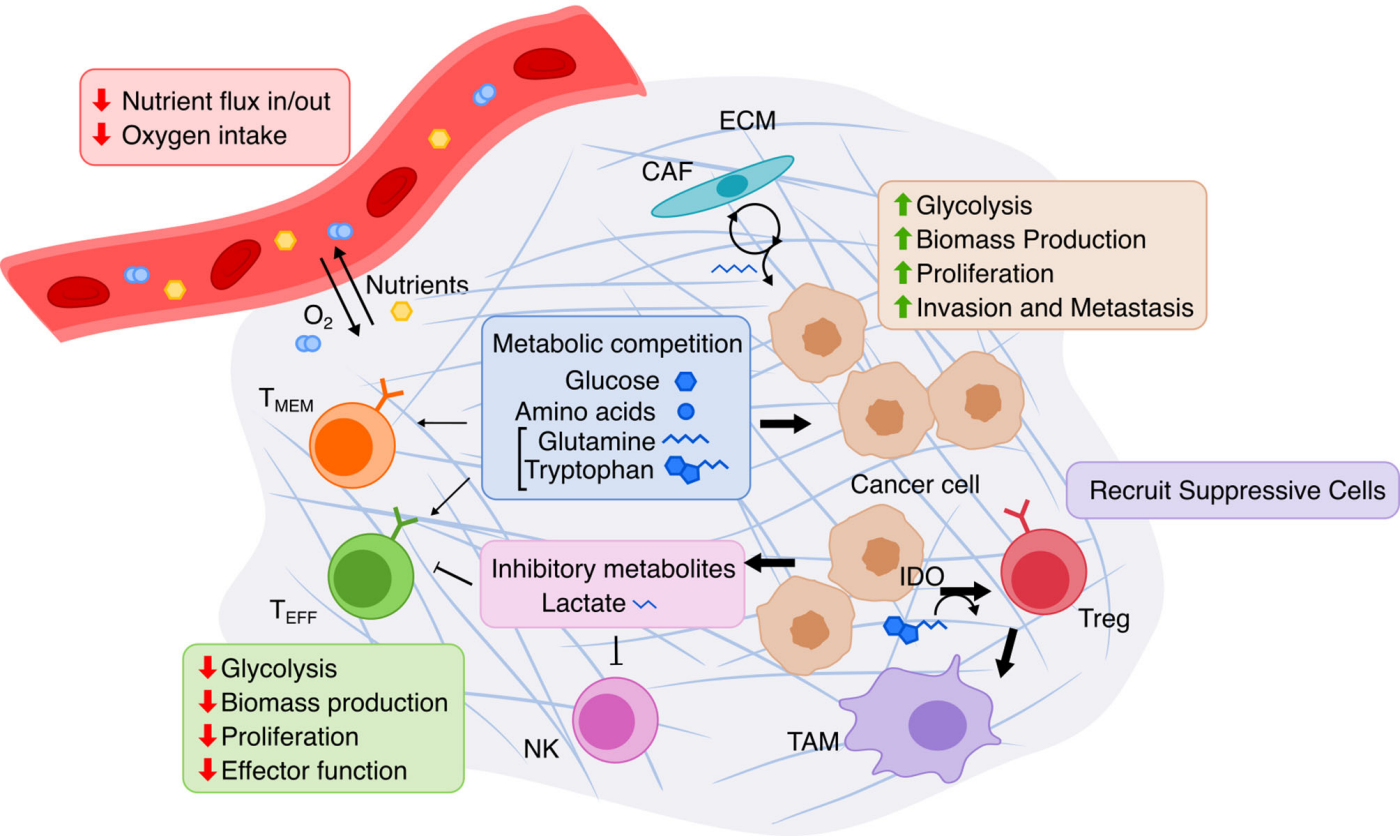
Overview of metabolic interactions within the tumor microenvironment. The TME is composed of cancer cells, immune cells, and stromal cells embedded in extracellular matrix (ECM). Limited nutrients and oxygen lead to metabolic competition between cancer and various lymphocytes, especially hampering anti-tumor activity of effector T cells (T_EFF_). Cancer cells adapts *via* upregulating nutrient transport and altering cancer-associated fibroblasts (CAF) to replenish metabolites. T cell immunity is further suppressed by cancer cells’ release of lactate produced by glycolysis and by recruitment of immune-suppressive cells due to Indoleamine 2,3-dioxygenase (IDO) activity. T_MEM_, memory T cell; NK, natural killer cell; Treg, regulatory T cell; TAM, tumor-associated macrophage.

The dramatic functional and environmental changes that occur during cancer formation and progression are accompanied by accordingly dramatic metabolic reprogramming in cancer cells (**Figure 3**). These changes canonically include the Warburg effect (132, 133), the switch from predominantly mitochondrial oxidative phosphorylation to aerobic glycolysis, potentially done to increase biomass production critical to maintain high proliferation (133); this leads to increased glucose uptake and lactate secretion by cancer cells. Increased energy and biomass production in cancer cells is also associated with increased uptake and synthesis of amino acids (134), fatty acids (135), and nucleotides (136). The TME is also quite distinct from normal physiology as it espouses a different set of spatial structures, nutrient/metabolite compositions, and cellular heterogeneities, and thus the metabolism of cancer cells is further perturbed just as the cancer cells metabolically influence the TME in turn (137). In the TME, tumor cells also inhibit immune cells by outcompeting them for critical nutrients with finite supply, such as glucose and amino acids, thereby limiting immune anti-tumor activity. The manifold metabolic changes that occur in cancer pose a challenging question to faithfully model. However, overcoming this challenge to establish an accurate model of this complicated metabolic reprogramming may prove useful for identifying potential targets, such as cell-cell metabolic interactions between tumor and immune cells, for cancer therapy.

**Figure 4:**
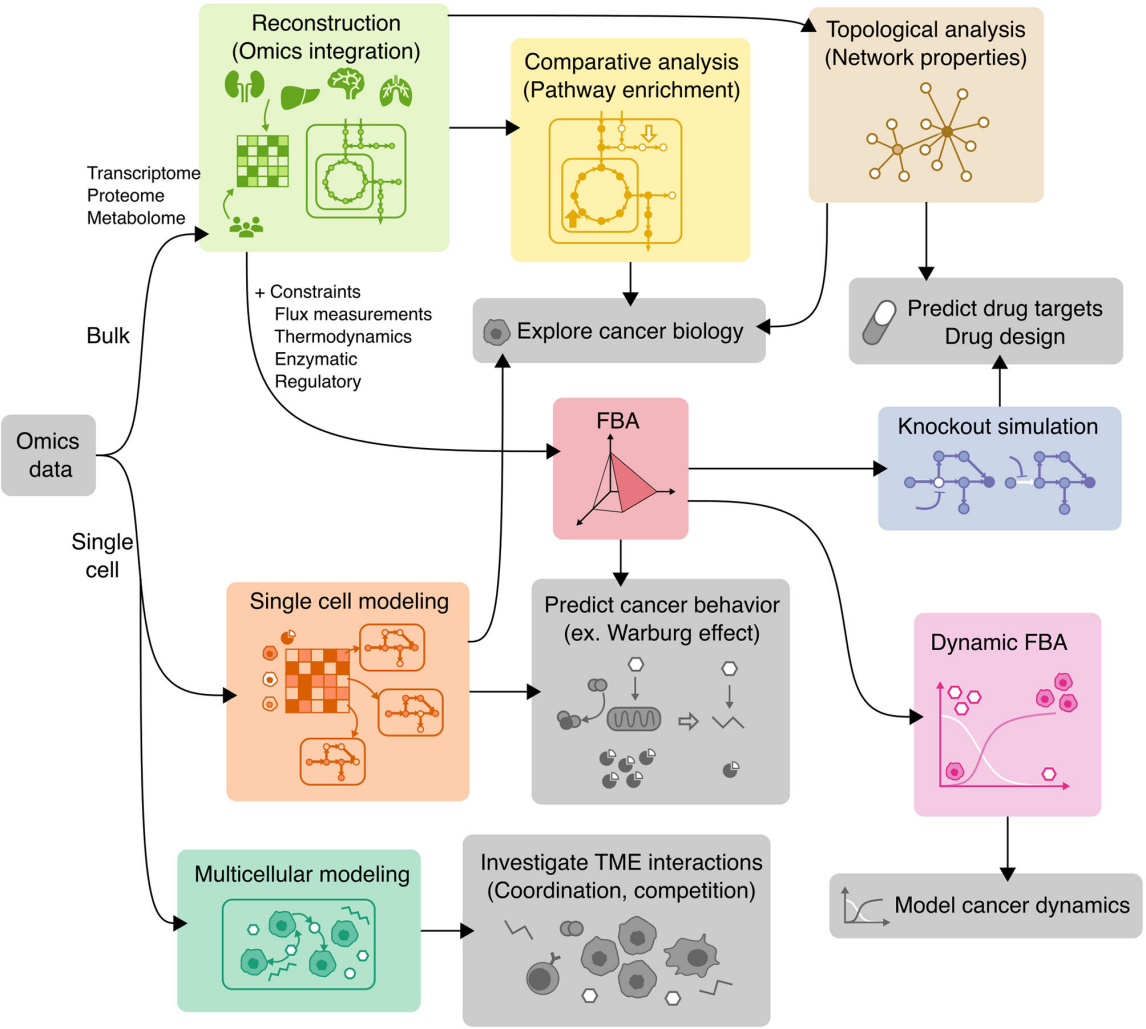
Applications of COBRA methods to cancer research. Workflow diagram of using various COBRA methods (colored) in combination to achieve different objectives (grey).

COBRA methods offer a way to computationally achieve this goal, such as inferring metabolic state *via* FBA which requires an objective function. While designing an objective function for tissue-specific eukaryotic cells is usually challenging, cancer cells can be reasonably modeled by biomass objective function, because cancer is mainly characterized by cellular growth (138). This makes flux predictions better suited for modeling cancer than healthy tissues, which do not actively proliferate. To simulate flux through cancer GEMs, studies have used objective functions representing growth as consumption of biomass precursors (139), or individual required metabolic tasks such as energy and redox, internal conversions, substrate utilization, biosynthesis, and biomass growth (54, 108). Some studies found gene-essentiality predictions from GEMs to be robust to definition of biomass composition (139) and capable of predicting growth kinetics in small-scale model (140), suggesting that the biomass equation is not significantly biased. However, another small-scale model claimed that elemental mode flux predictions using lactate objective is better than biomass objective at predicting experimental fluxes (141). These differences emphasize the importance of experimental validation to look for bias and sensitivity analysis to see if our biological insights are heavily affected by objective function definition and other system assumptions. Furthermore, the assumption that cancer cells optimize for cell growth may not always hold as tumors adapt, especially under selective pressure from therapies and immune system to adopt a quiescent state (138). Even if a proper objective is used, there are many optimal FBA solutions, and some may not be biologically viable due to inaccurate reaction bounds, violation of steady-state assumption, regulatory processes, and other limitations to our biological knowledge. Despite these limitations, past cancer applications of COBRA methods strived to improve our understanding of the disease and identify drug targets *via* comparative analysis, network analyses, quantitative flux simulations, and TME modeling. These studies have been reviewed multiple times (13, 14, 138, 142–144), and we have compiled the collection of these studies in **Table 3** and summarized their applications below (**Figure 4**).

**Table 3:** List of cancer metabolic modeling studies.

Ref.	Cancer	Purpose					Method					
		Drug design Predict drug targets Explore Cancer biology Explain Warburg effect Patient classification					Integration		Model	Analysis	Constraints	Objective	
(145)	Breast	x		x				Lee-12	HMR 1	FBA, Comparative, Topological	Transcriptome, Fluxomic	Data Correlation	
(146)	Colorectal	x		x				tINIT	Human1	TFA, TFVA, pTFVA	Thermodynamic, Transcriptome, Biomass	Biomass	
(147)	Eye	x		x				iMAT	Recon 2	Gap filling, FBA, FVA, Knockout	Transcriptome	Biomass, Tasks	
(148)	Head and Neck	x		x				Upper bound	Recon 2	FBA, Sampling, Knockout	Thermodynamics, Enzyme kinetics, Transcriptome, Metabolome	ATP, NADPH	
(149)	Liver	x		x				iMAT-like	Recon 1	Comparative, FBA, Sampling	Transcriptome	Data Similarity	
(150)	Multiple	x		x				tINIT	HMR 2	Comparative, Knockout	Transcriptome	Tasks	
(151)	Multiple	x		x				tINIT	HMR 2	FBA, Knockout	Transcriptome	Biomass, Tasks	
(140)	Generic	x			x				Small-scale	FBA, DFBA, FVA, Knockout, Sampling		Biomass	
(152)	Kidney	x				x		tINIT	iCancer-Core	FBA, Knockout	Transcriptome	Biomass	
(153)	Brain	x						tINIT	HMR 2	Comparative, FBA, Knockout	Transcriptome	Biomass, Tasks	
(105)	Breast, Lung, Multiple	x						Upper bound	HMR 1	FBA, Sampling, Knockout	Transcriptome	Biomass	
(139)	Generic	x						MBA	Recon 1	FBA, Knockout	Transcriptome	Biomass	
(154)	Kidney	x						MBA	Recon 1	FBA, Knockout	Transcriptome	Biomass	
(54)	Liver	x						tINIT	HMR 2	Comparative, FBA, Knockout	Proteome	Biomass, Tasks	
(155)	Liver	x						tINIT	HMR 2	FBA, Knockout, Topological	Transcriptome	Biomass, Tasks	
(156)	Multiple	x						iMAT	Recon 1	FBA, ML, Topological	Transcriptome	Data Similarity	
(102)	Multiple	x						GIMME	Recon 2	FBA, Sampling, Knockout	Mutations, Transcriptome	Biomass	
(157)	Prostate	x						tINIT	iCancer-Core	FBA, Knockout, Sampling	Transcriptome, Proteome	Biomass, Tasks	
(158)	Breast, Kidney, Liver, Prostate		x	x					KEGG	Network Propagation, Knockout, ML	Transcriptome		
(159)	Colorectal		x	x				tINIT	HMR 2	Comparative	Transcriptome		
(160)	Multiple		x	x					Recon 2	Regulatory, Topological, ML	Transcriptome, Metabolome		
(111)	Multiple		x	x				mCADRE	Recon 1	Comparative	Transcriptome	Tasks, Biomass	
(103)	Multiple, Brain, Lung, Breast, Leukemia, Prostate		x	x				tINIT	Recon 3D	Comparative, Knockout, ML	Mutations, Protein Structures	Biomass	
(161)	Prostate		x	x				iMAT	Recon 2	FBA, FVA	Transcriptome	Data Similarity	
(162)	Kidney, Prostate		x					INIT	HMR 1	Knockout	Proteome, Fluxomic	Biomass	
(108)	Multiple		x					INIT	HMR 1	Comparative	Proteome		
(163)	Multiple		x							Topological			
(164)	Generic			x	x				C2M2N	FBA		Biosynthesis, Biomass	
(165)	Breast, Colorectal			x		x			Recon 2	FVA, ML	Metabolome	Metabolite Change	
(166)	Liver			x		x		tINIT	HMR 2	Comparative	Transcriptome, Proteome	Biomass, Tasks	
(167)	Brain			x				GIMME, MADE	iMS570	pFBA, Sampling	Transcriptome	Biomass	
(168)	Breast			x				E-Flux	Recon2	FBA	Proteome	Biomass	
(169)	Colorectal			x					Recon 2.2	Comparative	Transcriptome		
(170)	Colorectal			x				CORDA	Recon 2.2	FBA, FVA, Topological	Proteome	Biomass, ATP	
(130)	Generic			x					HMRcore	popFBA, Sampling	Loopless	Biomass	
(171)	Kidney			x					Recon 1	pFBA	Flux measurements	Biomass	
(172)	Kidney			x				INIT	HMR 1	Comparative	Proteome		
(173)	Liver			x				tINIT	HMR 2	Comparative, Gap filling, Regulatory, FBA	Transcriptome, Metabolome	Biomass	
(174)	Lung			x						13C flux analysis	Flux measurements, Labeling measurements		
(141)	Lung			x					Central Carbon, Recon 2	Elementary modes, Structural fluxes, pFBA	Protein efficiency	Biomass, Biosynthesis	
(131)	Lung, Breast			x				E-Flux	HMRcore	scFBA	scRNA-seq, metabolomics	Biomass	
(175)	Lung, Prostate			x				E-Flux	Recon 1	FVA	Transcriptome	Biomass	
(176)	Multiple			x				tINIT	HMR 2	Comparative	Transcriptome		
(104)	Prostate			x				IMAT, GIMME, Gonçalves, MADE	HMR 2	FBA	Transcriptome	Data Similarity	
(177)	Generic				x				Recon 1	FBA, FVA, Sampling	Protein efficiency, Enzyme kinetics	Biomass	
(178)	Generic				x				ATP	FBA	Protein efficiency	ATP	
(179)	Generic				x				ATP, BiGG	FBA	Protein efficiency	ATP, Nutrient cost	
(180)	Liver				x			MADE	Recon 2	Comparative	Transcriptome	Data Similarity	
(106)	Liver				x			Upper bound	Recon 3D	FBA, FVA	Transcriptome, Nutrient availability	Biomass	
(181)	Liver				x			Bounds	Recon 2	FBA	Protein efficiency, Transcriptome	ATP	
(182)	Multiple				x			E-Flux	Recon 1	FBA	Transcriptome	Biomass	

### Reconstruction of Cancer Metabolic Models

To date, numerous efforts have iteratively improved reconstruction of the human metabolic network within the Recon series (Recon 1, 2, 3D) (103, 183, 184), the Human Metabolic Reaction (HMR) series (HMR 1 and 2) (185, 186), and their derived unified model Human1 (187) (**Table 4**). From these generic human GEMs, cancer-specific metabolic models were generated by integrating multi-omics data to reduce the number of reactions to reflect cancer-specific activity. To extract multiple healthy and cancerous tissue-specific GEMs, studies utilized protein levels from Human Protein Atlas along with INIT algorithm (108) or CORDA algorithm (170). Other studies constructed cancer GEMs using transcriptomic data from 1) cancer cell lines in combination with different integration algorithms such as MBA (139), tINIT (150), a likelihood-based method (156), PRIME (90), and FASTCORMICS (112), or 2) transcriptomic data from tissue samples in combination with mCADRE algorithm (111). While transcriptome measurements can capture more genes, its data is noisy and does not correlate well with protein levels (190). In contrast, proteomic data more directly corresponds to enzymatic activity, but was previously limited by antibody or spectrometry methods that are low-throughput and less quantitative. Emerging evidence shows that newly developed quantitative proteome may better explain genetic disease and metabolism (191), emphasizing the advantage of using proteome evidence for metabolic model reconstruction. However, accuracy of proteome-based reconstructions is still limited due to various regulatory mechanisms such as protein modifications that have yet to be integrated into cancer metabolic models.

**Table 4:** Human metabolic generic models and cancer models.

Model	Scale	No. of Reactions	No. of Metabolite	No. of Genes	Web link
HMR 1 (185)	Genome	8174	6006	3674	https://metabolicatlas.org/gems/repository/366
HMR 2 (186)	Genome	8181	6007	3765	https://metabolicatlas.org/gems/repository/367
Recon 1 (183)	Genome	3741	2766	1905	http://bigg.ucsd.edu/models/RECON1
Recon 2 (184)	Genome	7440	5063	2194	https://www.ebi.ac.uk/biomodels/MODEL1109130000
Recon 3D (103)	Genome	10600	5835	2248	https://www.vmh.life/#downloadview http://bigg.ucsd.edu/models/Recon3D
Human1 (187)	Genome	13069	8366	3067	https://github.com/SysBioChalmers/Human-GEM
Cancer central metabolism (140)	Small	80	66	46	https://doi.org/10.1371/journal.pone.0012383
iCancer-Core (iHCC2578) (151, 166)	Genome	7762	5566	2892	https://github.com/sysmedicine/phd2020/tree/master/GEM/data
C2M2N (164)	Small	77	54	–	https://doi.org/10.3390/metabo9050081
HMRcore (131, 188)	Intermediate	315	256	418	https://github.com/BIMIB-DISCo/scFBA
Central Carbon (141)	Small	114	120	–	https://doi.org/10.1042/bst20150149
iMS570 (brain) (189)	Genome	630	524	570	http://dx.doi.org/10.1016/j.fob.2014.05.006

This table describes various human reconstructions that are used as the starting reference models in various cancer applications listed in **Table 3**. The most updated online links to these models may be different than previously described in their original manuscripts.

In the past decade, many more cancer-specific models have been reconstructed for liver (106, 149, 155, 166, 173, 181), kidney (152, 154, 162, 172, 172), breast (103, 105, 131, 145, 168), prostate (103, 104, 157, 161, 162, 175), brain (103, 153, 167), colorectal (159, 170, 192), head and neck (148), eye (147), and lung (103, 105, 131, 175) cancer to generate cancer-specific hypotheses. To compare these various methods for reconstruction of cancer metabolic models, a study benchmarked their predictive performance and consistency (91), with relevant findings summarized in **Table 2**. In pursuit of personalized medicine to find optimal treatment based on patient’ genetic factors, researchers have also built personalized cancer GEMs from patient sample data to identify metabolic features that are commonly-shared or patient-specific (54). Furthermore, patient genetic variants were integrated in the Recon3D model with protein structures to look for cancer mutation hotspots in glioblastoma patients (103). Nam et al. modeled loss of function mutations *via* knockouts and analyzed potential gain of function mutations by adding promiscuous reactions predicted by chemoinformatics (102). Overall, these various reconstructions of cancer metabolic models aim to capture the heterogeneity of cancer.

### Pathway and Network Analyses of Cancer GEM

To find metabolic differences between cancer and healthy cell types and between patients, these reconstructed metabolic networks are analyzed for enrichment of biological features, generating biologically relevant hypotheses that can guide mechanistic interpretation, biomarker discovery, and drug development. Comparative analysis involves statistical testing for the enrichment of reactions, genes, and metabolites to identify differentially activated pathways. Comparing networks of healthy and cancer cell types using hypergeometric test identified enrichment of not only well-known drug targets (polyamines, isoprenoid biosynthesis, prostaglandins and leukotrienes), but also new drug targets explained by protection against oxidative stress and methylglyoxal toxicity (108). Another study that used Wilcoxon rank sum test to compare tumor and normal metabolic models also found enrichment of leukotriene synthesis in addition to other tumor supporting pathways such as folate metabolism, eicosanoid metabolism, fatty acid synthesis, and nucleotide metabolism (111). Of note, these pathways were not statistically significant from pathway analysis of gene expression data alone, emphasizing the importance of systems-level network analysis to extract biological signal. In addition, the presence and absence of active genes, metabolites, and reactions can be characterized by clustering to validate similarity of related cell types (108), and calculating Hamming distance or pairwise comparisons to find the most different cancer GEMs (150). Comparing cancer-specific GEMs can reveal cancer types with more severe metabolic dysfunction. For example, clear cell renal cell carcinoma (ccRCC) GEM showed loss of redundant genes in key metabolic pathways (162, 172), suggesting that ccRCC might be more responsive to metabolic anticancer drugs due to reduced capacity to evade drug inhibition *via* alterative enzymes and pathways.

While the presence of pathways is indicative of activity, analyzing the pattern of how these pathways connect could provide additional insights. For this purpose, topological analysis is a network-based analysis that characterizes metabolic models based on network properties that describes the degree and patterns of connection between metabolites, genes, and reactions. The same models from Agren et al. (108) were converted to enzyme-enzyme networks and re-analyzed using topological analysis, which revealed that most approved cancer drugs do not correlate with centrality (measure of importance) of individual enzymes, but do belong to a specific cluster in a cancer enzyme-centric networks (163). Furthermore, the analysis found that certain network motifs, such as feed-forward loop, are enriched in cancer networks compared to healthy cell type. Utilized in several other cancer studies (**Table 3**), topological analyses reveal insights about cancer based on the structure of cancer-specific metabolic networks without using flux simulations. Topological analyses emphasize the importance of system-oriented cancer drug design to find therapy that change the entire metabolic state instead of a single drug target that can be easily compensated by alternative pathways.

### Quantitative Prediction of Cancer Behavior

To better understand metabolic reprogramming within cancer cells, cancer-specific metabolic models were used to simulate flux distributions to illustrate their metabolic state. Initial efforts built generic small-scale cancer models that only included the major pathways in cancer such as ATP and biomass production (140, 178) to demonstrate the usefulness of standard COBRA methods as such FBA, FVA, and *in silico* knockouts (140). Performing dynamical FBA on such model was able to predict the growth rates of HeLa cells, validating the use of biomass objective with FBA for cancer predictions (140). While constraints on glucose uptake and solvent capacity initially predicted the Warburg effect (178), later implementations of protein constraints in these small-scale (179) and genome-scale (177) cancer models explained the Warburg effect as a result of maximizing enzyme efficiency. Another protein efficiency constraint, flux minimization with FBA, predicted the Warburg effect in liver-specific GEMs and agreed with metabolic profiling of *Mir122a* knockout mice (181). Another cancer metabolic adaptation that bypass mutation of enzymes from the TCA cycle was recapitulated by adding upper flux bounds during flux simulations (154). In addition to these methods for modeling intracellular constraints, it is also important to account for cell-extrinsic factors imposed by the tumor microenvironment. Approaches to impose nutrient constraints include constraining exchange reaction bounds by experimentally measured flux (145, 162), transporter expression (105), concentration and membrane potential-dependent free energy calculations (148), and concentration gradient over time (106). These quantitative predictions of cancer metabolic reprogramming further demonstrate the applicability of COBRA methods to model cancer metabolic programs.

### *In Silico* Drug Discovery

Furthermore, quantitative flux predictions can guide drug therapy design by simulating the effect of enzyme inhibition on cellular metabolic function in both cancer and healthy GEMs to maximize therapeutic effect while minimizing toxicity. *In silico* knockouts are performed by constraining one or more reactions’ flux to zero, setting an objective function that represents growth or other metabolic tasks, and finally performing FBA to calculate the change in maximum objective. One approach aims to find drug targets based on gene essentiality–knock out of enzymes that inhibit cancer growth. *In silico* gene knockout simulations of genome-scale cancer model identified drug targets and combination drug strategies (double gene knockout) that could reduce cancer growth (139). These candidates include known drugs and are validated *via* shRNA gene silencing data and cancer somatic mutations. Another study found that gene essentiality by FBA using biomass objective is better than chance but has limited accuracy depending on cancer type, especially after adding exchange flux constraints (162). A second approach based on metabolite essentiality screens for antimetabolites (metabolite analogs), which would compete with endogenous metabolites to inhibit their associated enzymes. By simulating *in silico* knockout of all enzymes acting on each metabolite, studies have identified antimetabolite drug candidates that could selectively disable critical metabolic task in cancer cell line-specific GEMs (150) and personalized hepatocellular carcinoma (HCC) patient GEMs (54). Out of 101 antimetabolite candidates, many were already used (22%) or proposed as anticancer drug targets (60%), and some targets were shown to be highly patient-specific, supporting the use of flux predictions of cancer GEMs for both general and personalized drug discovery (54). Many more studies applying *in silico* knockouts are listed in **Table 3**. While using FBA for *in silico* drug design is well established, the predictions maybe inaccurate due to bias introduced by the choice of objective function and reaction bounds, such as those for cell-specific exchange fluxes that are not always experimentally determined (91, 162). Furthermore, simulations based on cell line measurements and culturing conditions cannot faithfully reflect multi-cellular tissues and physiological environments *in vivo*.

### Multicellular and Single-Cell Modeling of TME

To analyze cell-heterogeneous systems like the TME, it is important to investigate metabolic programs within a multi-scale population model and at the single-cell level. To model interactions between multiple cells, multicellular modeling accounts for metabolite exchange between single cells within the environment. This was attempted by popFBA (130), which simulated a spatial model of identical cancer cells that adapted heterogeneously and cooperatively to maximize growth of the entire tumor mass. To account for tumor heterogeneity, a population model can be constrained by single-cell RNA-seq (scRNA-seq) data containing different tissue subpopulations in the scFBA method (131). When applied to lung adenocarcinoma and breast cancer cells, scFBA reveals metabolically defined subpopulations, some of which have coordinated metabolic fluxes (e.g., uptake or secretion of opposite sets of metabolites) suggesting potential cell-cell metabolic interactions. Other methods, such as scFEA or Compass, calculates cell-wise metabolic flux from scRNA-seq data to interpret cellular metabolic activity. Compass revealed metabolic states associated with functional states of T helper 17 (Th17) cells, in particular an increase in arginine and polyamine metabolism that resulted in a regulatory T cell (Treg)-like, dysfunctional cell state (73). The other single-cell method, scFEA, applied to patient-derived pancreatic cancer cells with metabolic perturbations (gene knockout, hypoxia), predicted flux variation that correlates with measured metabolomics. These methods could be applied to infer metabolic states of tumor and immune cells from existing scRNA-seq datasets of tumor samples. In future studies, algorithms for microbial community-modeling can be repurposed to investigate the interactions of cancer and immune cells in the TME (MICOM) and model the dynamics of immunosurveillance and tumor resistance (surfin_fba).

## Discussion

COBRA methods have proved useful for systems-level inference of metabolic activity under a mathematical framework built upon biomolecular knowledge. The accessibility and algorithms of COBRA methods have been improved with the development of open-source COBRA Python packages. We have identified Python packages available to handle the major areas of COBRA methods: FBA, FVA, gene knockout, strain design, omics integration, regulatory constraints, reconstruction, gap filling, ensemble modeling, thermodynamics, enzymatic constraints, EFM, sampling, single-cell modeling, multicellular modeling, and visualization. However, the Python COBRA ecosystem is currently missing some methods for constraining models by regulatory mechanisms and reconstruction of context-specific GEMs. However, these gaps are only due to limitations of time and effort, not limitations of the Python programming language. In fact, many features involving complex models, parallelization, and efficient memory management are available in Python instead of MATLAB. For example, ME-models, a set of multi-scale problems describing multiple biological processes across different space and time scales such as transcription, translation, and protein interactions, are handled by Python packages only for now. Integration of protein structure into the Recon3D human GEM was facilitated by Python packages ssbio and GEM-PRO (103). GEMs interface with machine learning in Medusa and scFEA. Likewise, upcoming COBRA packages will likely integrate with existing Python tools for statistical learning and analysis of single-cell multi-omics data. As models and omics datasets increase in complexity, COBRA methods will thrive in the open-source Python environment. While we improve our modeling techniques, it is also important to validate flux predictions using experimental techniques such as metabolomics profile and label tracing experiments. To interpret isotope tracing data, ^13^C-Metabolic Flux Analysis was developed to infer intracellular fluxes. While ^13^C-MFA allows direct measurement of metabolic flux, the method is limited to small-scale models (central metabolism) and requires more expertise than the typical omics measurements for constraining COBRA methods. Python packages for modeling label tracing data are available *via* FluxPyt and mfapy (193, 194). While these experimental techniques are outside the scope of this review, they have been reviewed previously for bulk, single-cell, and cancer applications (119, 195, 196). Another alternative computational metabolic modeling approach is parametric kinetic modeling, which mathematically describes enzyme activity involving regulatory mechanisms (17). While this paradigm may offer accurate prediction of perturbation outcomes, systems emergent properties (e.g., switches, oscillations, bistability), and non-steady state concentrations, scaling kinetic models to genome-scale metabolic models is a challenge due to the requirement for intracellular concentrations, kinetic parameters, and rate laws. DMPy attempts to overcome the challenge by incorporating thermodynamics constraints to infer missing kinetic parameters. Hybrid approaches combining kinetic modeling with constraints-based models may bring kinetic modeling closer to genome-scale.

Applications of GEMs and COBRA methods to cancer research have improved our understanding of how molecular mechanisms translate to cancer phenotype, aiding interpretation of multi-omics data and guiding drug designs that target cell metabolism at the systems-level. Metabolic models of cancer have evolved from small-scale models of essential pathways to genome-scale cancer-specific models, and they are now expanding to the realm of single-cell modeling. The computational resources required for numerous single-cell reconstructions and optimizations can be costly. Single-cell methods reduce complexity by pooling of reactions and similar cells and could benefit from ensemble modeling techniques that reduce a large number of models into ensemble objects. As demonstrated by bulk-level modeling, future single-cell modeling can improve prediction accuracy by incorporating constraints determined by multi-omics, thermodynamics, protein crowding and kinetics, genotype, and regulatory mechanisms. Furthermore, single-cell methods that estimate the metabolic flux of individual cells can be improved by integration of spatial information and inter-cell metabolic exchange to model crosstalk between cancer, immune, and stromal cells within the TME. By understanding the cancer-immune metabolic competition, we can design drugs that disrupt pathophysiologic interactions to enhance antitumor immune response and prevent evasion of immunosurveillance.

## Author Contributions

RN conceived the review, wrote the manuscript, and created visualizations. JL wrote the manuscript and created visualizations. YS and JH supervised and edited the manuscript. PB and CD edited the manuscript. All authors contributed to the article and approved the submitted version.

## Funding

This work was supported by the Andy Hill Cancer Research Endowment Fund (JH) and the Parker Institute for Cancer Immunotherapy (JH). YS is a Damon Runyon Quantitative Biology Fellow supported by the Damon Runyon Cancer Research Foundation (DRQ-13-22). YS was additionally supported by the Mahan Fellowship at the Herbold Computational Biology Program of Fred Hutch Cancer Research Center and the Translational Data Science Integrated Research Center New Collaboration Award at Fred Hutch Cancer Research Center and in part through a pilot fund from the NIH/NCI Cancer Center Support Grant P30 CA015704. PB acknowledges the support of 5U01AG061359-02 and 5U01AG061359-03 from NIA and 5R01HD091527-06 from NICHD. JL was funded by the Brotman Baty Institute Catalytic Collaborations Trainee Grant program.

## Conflict of Interest

JH is a board member of PACT Pharma and Isoplexis and receives support from Gilead, Regeneron and Merck.

The remaining authors declare that the research was conducted in the absence of any commercial or financial relationships that could be construed as a potential conflict of interest.

## Publisher’s Note

All claims expressed in this article are solely those of the authors and do not necessarily represent those of their affiliated organizations, or those of the publisher, the editors and the reviewers. Any product that may be evaluated in this article, or claim that may be made by its manufacturer, is not guaranteed or endorsed by the publisher.
